# Feline immunodeficiency virus: current insights into pathogenesis, clinical impact, and advances in treatment and vaccine development

**DOI:** 10.3389/fvets.2025.1665999

**Published:** 2025-09-25

**Authors:** Nahid Akhtar, Ragini Mishra, Shivakant Tripathi, Santiago Rendon-Marin, Manik Prabhu Narsing Rao, Andrés Felipe Cuspoca Orduz, Jorge Samuel Leon Magdaleno, Abdul Rajjak Shaikh, Julian Ruiz-Saenz, Luigi Cavallo, Mohit Chawla

**Affiliations:** 1 Department of Research and Innovation, STEMskills Research and Education Lab Private Limited, Faridabad, Haryana, India; 2 School of Bioengineering and Biosciences, Lovely Professional University, Phagwara, Punjab, India; 3 Grupo de Investigación en Ciencias Animales - GRICA, Facultad de Medicina Veterinaria y Zootecnia, Universidad Cooperativa de Colombia, Bucaramanga, Colombia; 4 Grupo de Investigaciones Biomédicas Uniremington, Programa de Medicina, Facultad de Ciencias de la Salud, Corporación Universitaria Remington, Medellín, Colombia; 5 Facultad de Ingeniería, Instituto de Ciencias Aplicadas, Universidad Autónoma de Chile, Centro de Investigación e Innovación, Huechuraba, Chile; 6 Grupo de Investigación en Epidemiología Clínica de Colombia (GRECO), Universidad Pedagógica y Tecnológica de Colombia, Tunja, Colombia; 7 Physical Sciences and Engineering Division, KAUST Catalysis Center, King Abdullah University of Science and Technology (KAUST), Thuwal, Saudi Arabia

**Keywords:** feline immunodeficiency virus, vaccine, prevalence, antiviral, retrovirus, symptoms

## Abstract

Feline immunodeficiency virus (FIV) is a retrovirus that infects both domestic and wild cats worldwide, causing a progressive decline in the immune function. FIV infection is a major concern for cat owners, particularly those with outdoor cats or multi-cat households, as it can lead to chronic illness and a reduced lifespan. The development of effective prevention and treatment strategies for FIV is therefore essential to improve the health and welfare of cats. This review article provides an overview of current knowledge on FIV, covering its epidemiology, prevalence, pathogenesis, risk factors, transmission, and management. It also discusses the various FIV subtypes, their geographical distribution, and their associations with different clinical outcomes. In addition, the review examines the clinical and pathophysiological features associated with FIV, including oral and respiratory infections, neurological disorders, renal diseases, and cancer. The review also discusses management strategies for FIV-infected cats, with a focus on advances in the development of antiretroviral drugs and immunomodulators. This review highlights the challenges of developing an effective FIV vaccine and provides a comprehensive summary of the latest advancements in FIV vaccine research. Additionally, it offers an overview of adjuvants used so far in FIV vaccine candidates and explores the potential application of adjuvants currently licensed for other vaccines. Overall, this review paper provides a comprehensive and up-to-date summary of current knowledge on FIV, highlighting key areas that require further research to improve treatment and prevention of this important feline viral infection.

## 1 Introduction

Feline immunodeficiency virus (FIV) is a lentivirus of the *Retroviridae* family, first reported in 1986 in California, USA, in domestic cats exhibiting immunodeficiency syndromes (1, 2). Since then, cases of immunosuppressive FIV infections have been reported in domestic cats worldwide (3–8). Beyond domestic cats, FIV has also been reported in bobcats, Pallas' cats, guignas, leopards, pumas, Tsushima leopard cats, and lions (9–15). FIV infection is established through the integration of a provirus, a DNA copy of the viral RNA, into the host genome, leading to lifelong infection (16). The course of infection is characterized by three main phases (17). The first, or the primary infection stage, occurs 3–6 weeks after infection, during which viremia develops and cats exhibit signs of anorexia, pyrexia, lymphopenia, neutropenia, peripheral lymphadenopathy, and malaise, lasting from weeks to months (18, 19). The second phase, the asymptomatic phase, is the longest and can persist for several years. During this period, viral replication occurs at minimal levels, and the cat remains clinically healthy. Notably, some cats may remain in this stage for their entire lifetime (7.5–12.5 years) (16, 20). The final stage is terminal infection, characterized by increased viral replication and the onset of clinical symptoms due to CD4+ lymphocytopenia (16, 18). Many cats infected with FIV can live as long as uninfected cats if provided with appropriate management and high-quality care in household settings. However, they remain predisposed to opportunistic infections and atypical diseases (21, 22). The infecting FIV subtype may also affect clinical outcome. One study reported that, although clinical symptoms and FIV subtypes were not significantly correlated, cats infected with subtype A viruses developed life-threatening conditions, including encephalitis and AIDS-like diseases.

In contrast, cats infected with subtype B viruses displayed either no symptoms or relatively mild manifestations, such as gingivitis and stomatitis (23). Similarly, subtype C has been reported to be more virulent than subtype A, with infected cats exhibiting a greater likelihood of lymphopenia and neutropenia in the first 10–12 weeks of infection. Cats infected with FIV-C also exhibited mean viral RNA levels up to 100-fold higher during the initial weeks of infection compared to those infected with subtype A. Furthermore, FIV-C-infected cats showed significantly elevated levels of proviral DNA in peripheral blood mononuclear cells, and proviral DNA levels in tissues, such as the popliteal lymph nodes, were approximately 10 fold higher at 20 weeks post-infection. These findings were accompanied by more severe histopathological lesions (24).

Since the discovery of FIV, numerous studies on both experimentally and naturally infected domestic cats have provided extensive knowledge regarding the virus, its prevalence, and the pathogenesis of the disease. Furthermore, many studies have been performed to develop vaccine candidates and antiviral drugs for the treatment of FIV. This review summarizes up-to-date information regarding FIV epidemiology, prevalence, and pathogenesis. Additionally, the review delineates the advances made in the search for therapies for the prevention and treatment of FIV in cats.

To ensure a comprehensive coverage of the literature, a systematic search strategy was employed. Relevant publications were retrieved from multiple databases, including PubMed, Web of Science, and Scopus, up to July 2025. The search was performed using the following keywords: “Feline immunodeficiency virus,” “FIV,” “feline immunodeficiency virus” OR “FIV.” Furthermore, the keywords “feline immunodeficiency virus,” or “FIV,” were used in combination with other keywords such as “pathogenesis,” “immune response,” “molecular mechanisms,” “diagnosis,” “treatment,” “vaccine,” and “management.” Some of the combined search terms are (“Feline immunodeficiency virus” AND “Vaccine”), (“Feline immunodeficiency virus” AND “Diagnosis”), and (“Feline immunodeficiency virus” AND “Treatment”). The inclusion criteria comprised (i) peer-reviewed research articles, reviews, and conference proceedings relevant to FIV biology, diagnostics, pathogenesis, and therapeutic approaches published in English and (ii) studies focusing on FIV in domestic cats or closely related felids. The exclusion criteria included studies not directly related to FIV (e.g., studies exclusively on HIV unless comparative) and non-English publications. Additional references were identified by manually screening the bibliographies of key papers.

## 2 Genome and molecular aspects of FIV

FIV is a positive-stranded RNA virus with a genome of approximately 9,400 nucleotides (25, 26). It contains three genes—gag, *pol*, and *env*—which encode the Gag protein, the pol polyprotein, and the envelope polyprotein, respectively (25, 27). The Gag protein is the precursor of structural proteins, including the matrix, capsid, and nucleocapsid proteins. It localizes and captures the viral genomic RNA for packaging within the host cell at the cytoplasmic face of the nuclear envelope (27, 28). In FIV, the packaging signal appears to consist of two parts: the first part spans the initial 250 nucleotides of the 5′ untranslated region, and the second part encompasses the start of the *gag* gene (27, 29, 30). The Y176/L177 motif in the C-terminal domain of the FIV capsid protein is important for viral infectivity, Gag assembly, and capsid oligomerization (31). Both the C-terminal and N-terminal regions of the capsid protein also contribute to Gag assembly (32). The deletion of the C-terminal p2 peptide of the Gag protein disrupts Gag assembly by eliminating the PSAP budding motif (32). The proximal zinc finger motif of the FIV nucleocapsid protein plays a more significant role in genomic RNA binding and virion production than the distal motif. This conclusion is supported by evidence showing that substituting serine for the first cysteine residue in the proximal zinc finger significantly impaired both genomic RNA binding and virion assembly.

In contrast, mutating the first cysteine residue in the distal zinc finger maintained significant RNA-binding activity in the mutant nucleocapsid protein and had no impact on virion production (33). The Pol polyprotein is cleaved into protease, reverse transcriptase, integrase, and deoxyuridine triphosphatase (26, 34, 35). FIV protease, which is an aspartyl proteinase, cleaves the gag-pol polyproteins during maturation into their respective functional structural proteins and enzymes, along with two other peptides, namely spacer peptide p1 and C-terminal p2 peptide (26).

FIV reverse transcriptase initiates the conversion of the single-stranded RNA genome into double-stranded DNA, a process believed to be regulated by interactions between the extreme 5′ nucleotides of the tRNA primer and a conserved stem-loop in the U5 inverted repeat region (27, 36, 37). Integrase is essential for the insertion of FIV proviral DNA into the host cell genome, with the N-terminal (residues 1–52) and C-subterminal domains (residues 189–235) necessary for 3′-end processing and strand-joining reactions (27, 38). Deoxyuridine triphosphatase functions to lower the concentration of dUTP by converting it to dUMP, which can then be used to synthesize dTTP. This prevents the incorrect insertion of uracil during reverse transcription and thereby reduces the likelihood of mutations in the viral genomic DNA (26, 27). The envelope polyprotein gives rise to surface and transmembrane glycoproteins that mediate FIV attachment and entry into host cells by binding to the CD134 and CXCR4 receptors (39, 40). The envelope glycoprotein gp36 facilitates fusion between FIV and host cells (41). In addition, the envelope protein contains a 175-amino-acid signal peptide at the N-terminus that enables evasion of tetherin, a host restriction factor that inhibits the release of FIV from infected cells (42–44).

Furthermore, the V5 loop of the envelope polyprotein plays an important role in determining whether FIV will be neutralized by virus-neutralizing antibodies in cats (27, 45). Additionally, it has three ORFs: Vif (viral infectivity factor), ORF 2, and Rev. ORF2, which is also known as OrfA, has a role in virion dissemination, transcriptional activation, cell cycle arrest of infected cells, and splicing control (27, 46, 47). Vif is essential for FIV replication (48) and counteracts the activity of apolipoprotein B mRNA-editing catalytic polypeptide 3 (APOBEC3), a feline restriction factor that inhibits FIV viral replication, through a ubiquitin/proteasome-dependent pathway (26, 49). The degradation of APOBEC3 is mediated by the interaction of FIV Vif with elongin B, elongin C, and cullin, which together form an E3 ubiquitination complex (50). In addition, the FIV protease could antagonize APOBEC3 by cleaving it within nascent virions (51). The Rev protein facilitates the export of unspliced and partially spliced FIV RNAs to the cytoplasm (26). Within Rev, amino acids 84–99 contain the nuclear localization signal, whereas amino acids 82–95 form the nucleolar localization signal (52).

## 3 Genomics and evolution of FIV

**Figure 1:**
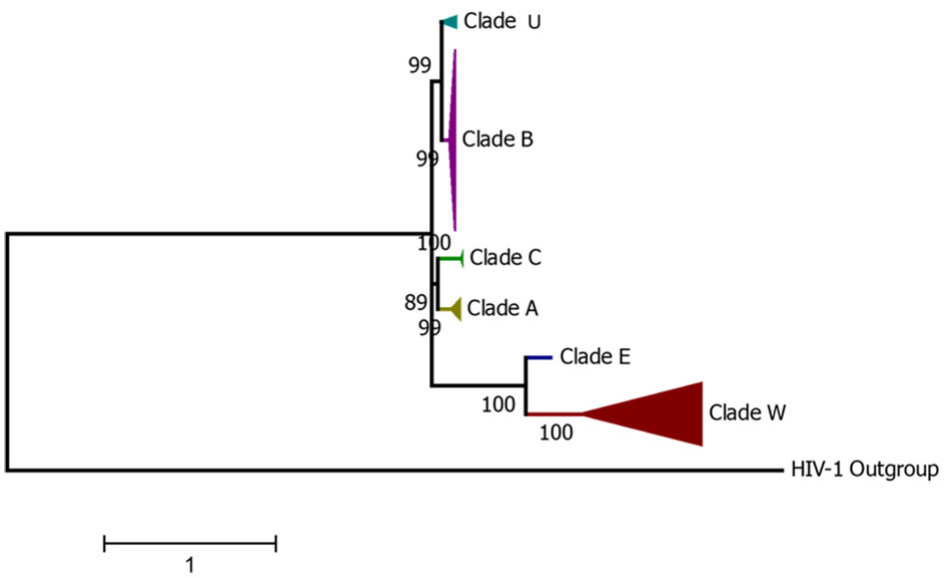
Condensed phylogenetic tree constructed from the alignment of complete FIV genomes from GenBank representing all current FIV-described Clades.

Based on the diversity of the V3–V5 region of the env gene, FIV can be classified into six clades (A to F) (23, 53–55). A seventh subtype named U-NZenv has been reported to be regionally distributed only in New Zealand (56, 57). Recently, a molecular study in Egypt proposed a novel FIV subtype, FIV-X-EGY, after phylogenetic analysis of env and gag sequences from infected cats showed that Egyptian strains form a distinct clade, genetically divergent from all known subtypes but with low internal variability and no evidence of recombination (58). Apart from the env gene, the FIV gag gene can also be used for the differentiation into different clades (59, 60). The nested PCR-restriction fragment length polymorphism analysis of a 329-base pair fragment within the FIV gag gene enabled the differentiation of FIV isolates belonging to subtypes A, B, and D, previously classified based on the V3–V5 region of the env gene sequence (60). The phylogenetic analysis of the full genome and the *env* gene allows the identification of at least six of the seven FIV subtypes (Figure 1), highlighting the high viral diversity among the sequences reported in GenBank. It is important to note that, similar to other retroviruses, FIV evolution is strongly driven by recombination and mutational events (61). A recent study analyzed 60 whole genome sequences (WGS) from the NCBI GenBank and discovered that the majority of recombination events (75%) occurred between wild-type host sequences within similar genomic regions, primarily located at the ends of the *pol, ORF1, ORF2*, and *env* genes [60]. In addition, both intra- and inter-subtype recombination events have been observed between the most prevalent FIV subtypes A and B (62, 63), and intra-host viral quasispecies have been reported, collectively contributing to the characteristics of the viral population and increasing viral diversity (64).

**Figure 2:**
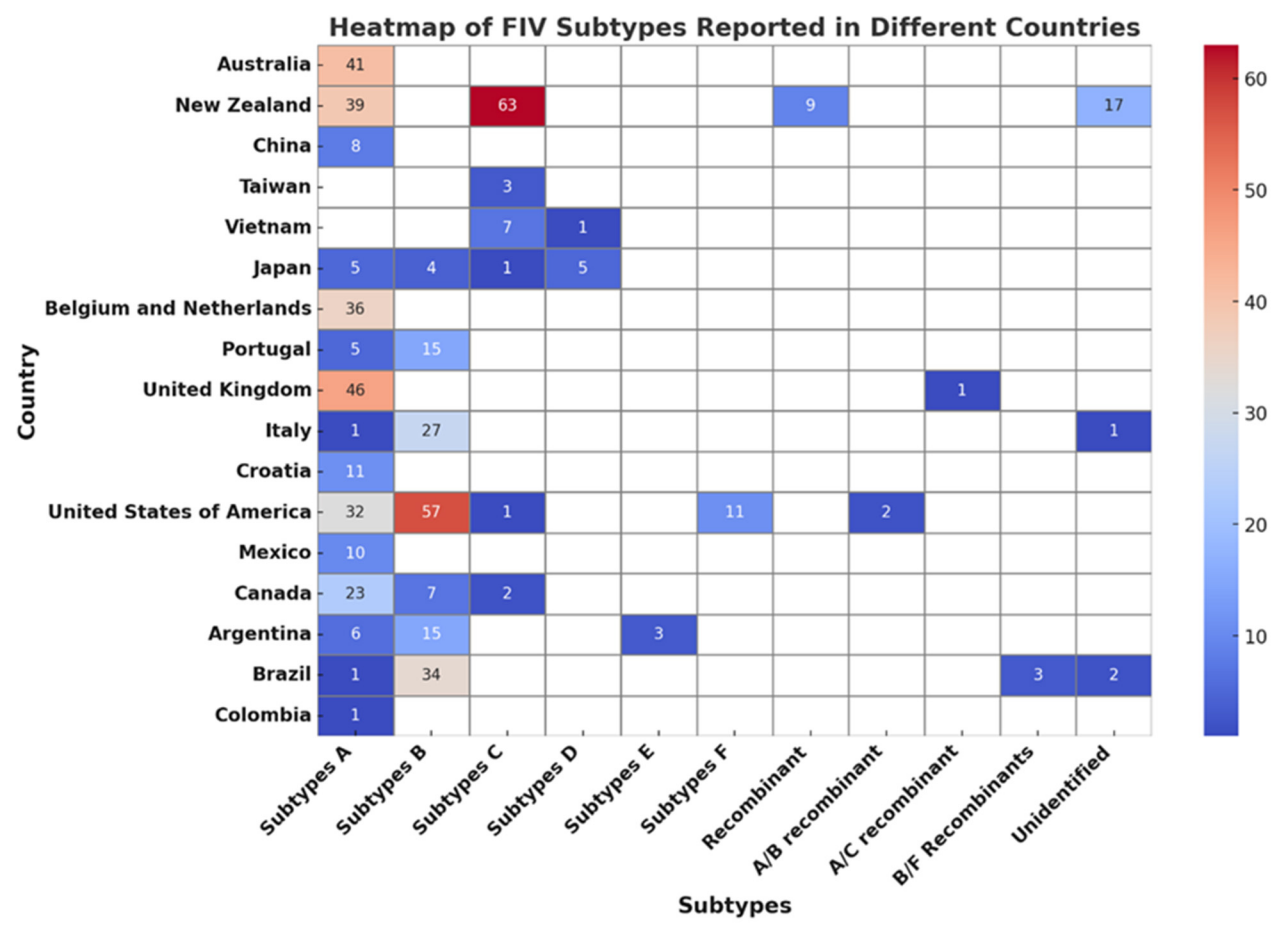
Heatmap analysis of FIV subtypes across different countries.

Among these seven subtypes, subtypes A and B are the most widely distributed, while the others are regionally spread (65). Recombinant FIV, such as subtype A/B recombinant and subtype B/F recombinant, has also been reported in North America and South America (66–68). Studies conducted in Brazil and Argentina have shown that subtypes B and E of FIV are present in South America, as revealed through the examination of either partial or complete genome sequences (66, 69). Additionally, studies conducted in Brazil and Colombia have identified subtype A of FIV (66, 70). Subtype D has been reported in Japan and Vietnam (71, 72). Figure 2 shows the subtypes of FIV reported in different countries.

## 4 Prevalence of FIV

FIV is found worldwide and exhibits higher seroprevalence in certain regions (7). A recent study found that FIV prevalence typically ranges between 5% and 8%, with a global average of 4.7% (73). The study further reported that the prevalence of FIV ranged from 2.19% to 23% in North America, 2.2–8.8% in Central America, 3.1–27% in Europe, and 3–22% in South America (73). A meta-analysis further revealed seropositivity rates of 5.93% in North America, 8.98% in Europe, 9% in Africa, 9.43% in South America, 10.9% in Central America, 11.9% in Oceania, and 14.34% in Asia (7). The rate of FIV infections in cats has been reported to be 15–16% for adult pet cats with outdoor access in Australia, where subtype A is more prevalent (4, 74). In European countries such as Germany and Ireland, FIV prevalence rates of 3.2% and 10.4%, respectively, have been reported (5, 75). The prevalence of FIV in different countries throughout the world is listed in Table 1. Based on the data from serological tests in Colombia, the prevalence of FIV has been determined to range from 6.7% to 13% (70). Whether the cats were healthy or unhealthy could also affect the prevalence rate of FIV. In a seroprevalence study of FIV in Canada and the USA, the overall seroprevalence was 3.6% but in cats infected with oral disease, respiratory disease, or bite wounds or abscesses, the seroprevalence was 9.7%, 6.4%, and 12.5%, respectively (76). A study indicated that FIV disease is more widespread among cats displaying aggressive behavior. Additionally, the aggressive FIV-infected cats were found to be more prone to having an unhealthy status when compared to their non-aggressive FIV-infected counterparts (77). Moreover, it has been reported that stray cats have a higher FIV seroprevalence (17.8%) than domestic cats that were relinquished by their owners (7.5%) (78). Additionally, FIV is more prevalent in older male cats (73).

**Table 1:** Prevalence of FIV in some countries.

**Country**	**Prevalence**	**Health status of cats studied**	**Method used for prevalence detection**	**Subtypes of FIV detected**	**References**
Algeria	32.39% of cats sampled from private veterinary clinics	Healthy: 56.33%, Sick: 43.66%	Immunochromatography analysis	Not mentioned	(216)
Argentina	21.45% by immunochromatography and 20.34% by nested PCR in domestic cats	All cats showed clinical signs of FIV	Immunochromatography and nested PCR	Not mentioned	(86)
Australia	12% in domestic cats	Not mentioned	FIV antibody testing kit	A (11/25), A/F recombinant (9/25), D/F recombinant (4/25), and F (1/25)	(168)
Australia	6% of cats were surrendered to the shelter	Not mentioned	Commercially available ELISA kit	Not mentioned	(74)
Australia	14.6–15% in client-owned cats	Not mentioned	Commercially available ELISA kit	Not mentioned	(74, 91)
Brazil	6.1% in client-owned domestic cats	Not mentioned	Detected using a commercially available ELISA kit, which was further confirmed by PCR	Only B	(217)
Brazil	6% in client-owned cats and 6.7% in stray cats	All were asymptomatic	PCR	Not mentioned	(218)
Brazil	7.65% in client-owned cats	Healthy (33%) and sick cats (67%)	Commercially available ELISA kit	Not mentioned	(121)
Brazil	23.3% in client-owned cats	Healthy (66%) and sick (33%)	Commercial immunochromatographic kit	Not mentioned	(219)
Belgium	18.8% in stray cats	Good health status (94.72%) and sick (5.28%)	Commercial immunochromatographic kit	Not mentioned	(95)
Canada	2.2% in shelter cats	Not mentioned	Commercially available ELISA kit	Not mentioned	(220)
Canada	5.5% in client-owned cats	Not mentioned	Commercially available ELISA kit	Not mentioned	(90)
China	1.5% (if client-owned or stray, not mentioned)	All clinically diseased	PCR	Not mentioned	(221)
China	1.3% in a mix of stray and client-owned domestic cats	A mix of healthy cats and cats showing clinical signs. Percentage not mentioned	FRET-PCR	Only subtype A	(55)
Czech Republic	5.8% domestic cats	Not mentioned	Commercial FIV gp40 antibody detection kit	Not mentioned	(222)
Egypt	31.7% in a mix of client-owned and shelter-housed cats	Not mentioned	Commercial antibody detection kits and PCR	Not mentioned	(58)
Greece	9.2% in a mix of stray and client-owned domestic cats	Not mentioned	Commercially available ELISA kit	Not mentioned	(87)
Germany	3.2% in client-owned cats	Not mentioned	Commercially available ELISA kit	Not mentioned	(5)
Hungary	9.9% (ELISA) and 13.1% (PCR) in client-owned domestic cats	Healthy (40.6%) and sick (59.4%)	ELISA or PCR of the pol gene	Only B	(65)
Ireland	10.4% (ELISA) and 9.3% (PCR) in client-owned domestic cats	Healthy (46.45%) and unhealthy (53.55%)	ELISA or PCR of the pol gene	A (7/8 samples), B (1/8 sample)	(75)
Japan	23.2% in client-owned domestic cats	66.38% exhibited clinical signs, and the remaining had no clinical signs	Commercially available ELISA kit	The most prevalent FIV subtype was B (42.2%) followed by A (30.2%), D (22.1%), and C (5.5%)	(223)
Lebanon	18.84% in household domestic cats	Healthy: 42.69%, Sick: 57.31%	Commercially available ELISA kit	Not mentioned	(224)
Malaysia	10% in domestic cats	Not mentioned	Commercially available ELISA kit	Not mentioned	(92)
Malaysia	31.3% domestic cats (115/368)	Healthy: 9/178 (5.1%), Sick: 36/190 (18.9%)	Commercial immunochromatographic kit	Not mentioned	(225)
Mozambique	11.0% in household cats	Healthy (66.9) and sick (33.1)	Commercially available ELISA kit	Not mentioned	(3)
Namibia	1.43% in domestic cats	Not mentioned	Polymerase chain reaction	Not mentioned	(226)
Namibia	4% in domestic cats	Not mentioned	ELISA	Not mentioned	(227)
New Zealand	13.7% in cats at the shelter house, consisting of strays and cats relinquished by owners	Good (47.4%), average (28.09%), and poor (23.2%)	Commercially available ELISA kit	Not mentioned	(78)
New Zealand	18.5% in client-owned cats	Not mentioned	Commercially available ELISA kit	Not mentioned	(93)
Poland	4.3% in household cats	All cats were suspected of infectious diseases	Immunochromatography	Not mentioned	(228)
Portugal	10.2% in stray cats	Not mentioned	Immunoblotting	Not mentioned	(229)
Russia	1.06% in client-owned cats	All sick	PCR	Not mentioned	(230)
Serbia	23.6 in domestic cats	Mix of healthy (71.2%) and ill (28.8%) cats	Commercially available ELISA kit	Out of 36 samples whose phylogeny was determined, 24 were subtype D, and 9 were subtype F. One was subtype A, one was subtype B, and one was undesignated.	(231)
Spain	7.87% in stray cats	All cats were seemingly healthy	Commercially available immunochromatographic kit	Not mentioned	(232)
Thailand	5.8% in client-owned domestic cats	All healthy	Commercially available ELISA kit	Not mentioned	(6)
Thailand	8.3% in client-owned domestic cats	Not mentioned	Commercially available ELISA kit	Not mentioned	(88)
Thailand	24.5% of pet cats (183/746)	Not mentioned	Commercial ELISA kit	Not mentioned	(233)
Thailand	2.67% of the domestic cats from animal hospitals	Not mentioned	Polymerase chain reaction	Not mentioned	(234)
Turkey	10.5% in domestic cats	Healthy or asymptomatic (83.5%), sick (16.5%)	PCR amplification of env and gag genes	B	(235)
Turkey	25.2% in stray cats	Not mentioned	PCR amplification of the gag gene	A, B, and C subtypes were detected	(236)
United Kingdom	9.5% in shelter cats	Not mentioned	Commercially available ELISA kit	Not mentioned	(237)
Vietnam	None (0/69 domestic cats)	Not mentioned	Indirect immunofluorescence assay and/or two commercial kits for FIV antibody	Not mentioned	(238)
USA	5.5–6.4% in feral cats	Not mentioned	Commercially available ELISA kit	Not mentioned	(239)
West Indies	17.1% in feral cats	Not mentioned	Commercially available ELISA kit	Not mentioned	(240)

## 5 Infection and transmission of FIV

The transmission of FIV occurs primarily through blood during bites (17). In households with socially well-adjusted cats, the likelihood of transmission is minimal. However, transmission can occur from an infected mother to her kittens, particularly if the mother is experiencing a severe infection. Transplacental transmission can occur in FIV-infected queens, but not all kittens in a litter may be infected. The overall rate of this type of transmission in the first year of infection is approximately 70% (79). The risk of mother-to-kitten transmission is higher in queens with a CD4+ count of less than 200 cells/μL, those showing signs of immunodeficiency, and those who contracted the virus within the last 15 months (80, 81). FIV-infected queen cats could also disseminate the virus to offspring via milk (82). Cats with FIV remain persistently infected, even though they can produce both antibody and cell-mediated immune responses (22). There is also a possibility of sexual transmission of FIV, as cell-free FIV has been detected in semen of naturally and experimentally FIV-infected cats (83). There is a possibility of transmission of FIV from naturally infected cats to uninfected cats in mixed households, but there are conflicting reports and a lack of evidence to support it. A study reported that, despite cohabiting the same household for years, there was no evidence of FIV transmission from infected cats to uninfected cats (84). Experimental infection of FIV in cats has shown that other methods of transmission, such as oral-nasal and rectal/vaginal mucosal transfer, could also be effective for transmitting the virus (85).

## 6 Risk factors of FIV infection in cats

Adult male cats with outdoor access are at a higher risk of FIV infection (86). Older cats are more frequently infected with FIV, likely due to its prolonged incubation period. During this time, cats can remain in an asymptomatic phase for several years, with minimal effects on morbidity and mortality rates (87). A study reported that male cats are four times more likely to test positive for FIV compared to female cats (87). Other studies have also reported that male cats are more susceptible to FIV infection (3, 76, 88). From a behavioral standpoint, male cats exhibit higher levels of aggression compared to female cats, which increases their risk of sustaining bite wounds and subsequently enhances the likelihood of FIV transmission (87). Another study reported that male urban feral cats exhibiting bold behavior have a higher probability of FIV infection (89). Cats with aggressive behavior are more likely to be infected with FIV (77). Bite wounds, oral diseases, and lethargy are significantly associated with FIV infection (90). Furthermore, mixed-breed and domestic-breed cats are also at higher risk of FIV infection than purebreds (91–93). The low prevalence of FIV among purebred cats may be attributed to their tendency to be kept indoors in smaller groups and their higher likelihood of being vaccinated (92).

Another study found that the American Wirehair and Persian breeds were less susceptible to FIV infection than domestic shorthair cats (88). It has been reported that neutered cats are less likely to be infected with FIV (74, 88). However, other studies have reported that neutered cats are more likely to be FIV-infected (94, 95). Feline leukemia virus co-infection could increase the risk of FIV as well (5). Cats living in multi-cat households are at higher risk of FIV infection than those in single-cat households (88). However, a previous study did not show any evidence to corroborate FIV transmission from naturally FIV-infected cats to non-infected cats in a mixed household (84). FIV transmission among cats sharing the habitat is less common unless they fight (84, 96). Symptoms such as weight loss, skin lesions, and/or pruritus, hyperglobulinemia, and gingivostomatitis are also associated with FIV seropositivity (87).

High testosterone levels have also been reported to be significantly related to FIV infections in cats (97). Aggressiveness in cats is known to be mediated by testosterone, which aligns with the findings showing a higher rate of FIV infection in males with elevated testosterone levels (97). Reduced levels of red blood cells and an albumin-to-globulin ratio below 0.6 are also associated with FIV infection (98). Cats in low socioeconomic status areas are at a higher risk of FIV infection (94). Cats infected with FIV are 1.6–2.3 times more likely to reside in areas of low socioeconomic status. This increased prevalence may be attributed to limited awareness of pet healthcare and a lower willingness or ability to invest in preventive measures, such as vaccinations, among residents in these areas (94).

## 7 Clinical and pathophysiological features of FIV

Typically, cats infected with the virus do not display any noticeable clinical symptoms for several years, and the development of the disease may be influenced by the strain of the virus that has caused the infection. In some cases, cats may not exhibit any symptoms at all (80). The most common clinical signs of FIV infection are secondary infections and immunodeficiency, resulting from a decrease in CD4^+^ cells, immunological anergy, and cytokine dysregulation (22, 80, 99). Secondary infections with *Toxoplasma gondii, Cladosporium carrion*, and *Leishmania infantum* have been reported in FIV-positive cats (100–102). Similarly, FIV-associated immunodeficiency has also been reported to facilitate parasitic infections such as *Eucoleus aerophilus* and *Cytauxzoon* sp. in cats (103, 104). Common symptoms of FIV include stomatitis, weight loss, lethargy, peripheral lymphadenopathy, mild fever, and chronic rhinitis (20, 22, 99). Depending upon the duration of infection, FIV can cause different hematological changes, such as anemia, leukopenia, eosinopenia, lymphopenia, pancytopenia, thrombocytopenia, hypochromia, hyperglobulinemia, and neutropenia in cats (4, 105, 106). FIV-infected cats have higher gamma-globulin concentrations due to increased antibody production by B cells (known as B cell expansion), triggered by the direct and indirect effects of the virus, including altered cytokine production and activation of specific T cell populations (4, 107).

FIV infection can lead to neurological abnormalities, including impaired motor function and cognitive deficits, in both naturally and experimentally infected cats (108, 109). These effects are associated with neuronal loss in the parietal cortex and hippocampus, as well as reduced glutamate receptor expression, which correlates with viral load and neuroinflammation (109). FIV infection may also act synergistically with age-related cognitive impairment, further exacerbating neurocognitive dysfunction in older infected cats (110). Reported behavioral neurological abnormalities in FIV-infected cats include facial and tongue twitching, delayed pupillary reflexes, psychotic behavior, sleep disturbance, and ataxia (99, 111, 112). A recent study has shown that the FIV glycoprotein gp95 increases Alzheimer's disease-related cellular tau pathology through cGMP-dependent kinase II (113). FIV-infected cats are five times more likely to develop tumors than non-infected cats (99). Among these, malignant lymphoma is the most common, although the underlying mechanism remains unclear (114). FIV is believed to contribute to lymphomagenesis primarily through indirect mechanisms, such as defective cell-mediated immunity or chronic lymphocyte activation. Direct involvement has been reported only once, in a case of clonal integration of the FIV genome (99, 114–116). Other tumor types observed in FIV-infected cats include fibrosarcoma, mast cell tumor, leukemia, and squamous cell carcinoma (80, 99, 117, 118). In addition, FIV can cause hyperglobulinemia and, in rare cases, bone marrow suppression (99, 119). Renal damage has also been associated with FIV infection, including glomerulonephritis, glomerulosclerosis, mesangial widening, and both interstitial and glomerular amyloidosis (120).

A study reported a case of plasma cell pododermatitis in a cat co-infected with FIV and feline leukemia virus, presenting clinical symptoms such as erythematous swelling of the paw pads, skin peeling, and alopecia (121). FIV has been linked to myocarditis, inflammatory myopathy, and hypertrophic cardiomyopathy in cats, with evidence of FIV infection detected in inflammatory cells within the myocardium (122, 123). FIV-infected cats have been reported to have significantly lower serum 25-hydroxyvitamin D concentrations than healthy control cats, similar to the lower serum levels of vitamin D in HIV-positive patients (124). FIV-infected cats have been reported to have a higher urinary protein-to-creatinine ratio and serum creatinine than healthy cats (125). Recently, FIV co-infection with *T. gondii* and *Mycoplasma hemominutum* has been reported to be associated with hemophagocytic syndrome in cats (126). A statistical association between FIV infection and infection with *Mycoplasma hemofelis or Mycoplasma hemominutum* has also been described, but it is not clear if FIV infection is a true risk factor for hemoplasmosis in cats (127, 128).

Considering the biology and clinical signs of FIV, FIV infection can lead to a progressive disruption of immunological functions in cats, similar to the pathogenesis of HIV, involving several key mechanisms and clinical manifestations (80). Hematological abnormalities have been reported since cats infected with FIV frequently exhibit lower levels of red blood cells (RBCs), hemoglobin, hematocrit, lymphocytes, and platelets in comparison to uninfected cats (98). Moreover, neutropenia is frequently observed in cats infected with FIV, as well as anemia and thrombocytopenia, compared to cats that are not infected (107). On the other hand, FIV infection results in changes to cytokine profiles, notably with elevated levels of interleukin-10 (IL-10) and interleukin-12 (IL-12) in treated cats, indicating modulation of the immune response. In contrast, untreated cats show a significantly lower IL-10/IL-12 ratio, which suggests a shift toward a more inflammatory immune response (129).

As expected, relevant clinical findings in infected cats have been proposed since FIV leads to a gradual reduction in CD4+ T lymphocytes, resulting in immunodeficiency and heightened vulnerability to secondary infections and neoplasia. Additionally, proteinuria is more frequently observed in FIV-infected cats, affecting 25% of infected individuals compared to 10.3% in non-infected cats (130). Furthermore, neurological symptoms and neoplasia, especially lymphoma, are frequently observed in FIV-infected cats (4). While FIV-infected cats have a higher likelihood of developing lymphoid malignancies, the association is less pronounced compared to feline leukemia virus infection (131).

Distinguishing FIV-related pathology from the comorbidities of aging in cats remains a major clinical challenge, since many conditions can occur independently of viral infection. Differentiating FIV-related pathology from age-associated comorbidities requires a multifaceted diagnostic approach. In naturally infected cats, a progressive inversion of the CD4^+^/CD8^+^ ratio (33) and elevated proviral load (34) are strongly associated with immune decline and clinical disease, whereas normal aging may not show these immunologic shifts. Quantitative molecular markers—viral RNA or integrated proviral DNA—have substantial potential for confirming active infection rather than attributing symptoms to age alone, which can be tested using PCR-based assays (35).

Neurologic and cognitive deficits, which may manifest subtly, are often overlooked in older cats unless specifically assessed. Opportunistic infections or neoplasms should be interpreted in the context of immunological indicators (CD4^+^/CD8^+^ T-cell ratios, lymphopenia, and hyperglobulinemia) and risk factors (outdoor access, fighting behavior, known FIV exposure), rather than attributed to aging alone. Monitoring immune and viral markers over time can therefore help clinicians distinguish FIV-driven pathology from coincidental age-related disorders.

## 8 Cellular and tissue reservoirs of FIV

FIV infects various cell types. For entry and infection, FIV targets CD4+ cells by binding its major surface glycoprotein to the CD134 receptor present on CD4+ cells, causing depletion of CD4+ cells (40, 80, 132). FIV can also infect CD8+ T cells by binding to CXCR4 receptors (40). In the later phase of FIV infection, it can also affect B cells, with a study reporting that the FIV provirus was most abundant in B cells in cats infected for more than 5 years (40, 133). FIV has been reported to infect and activate CD4^+^CD25^+^ regulatory cells throughout the acute phase of infection (134). CD4^+^CD25^+^ regulatory cells negatively regulate the immune response by inhibiting the proliferation and causing apoptosis of activated CD4^+^ and CD8^+^ cells (80, 135). FIV is also capable of infecting other leukocytes, including monocytes and dendritic cells (136, 137). In monocytes, adherence induces the expression of viral antigens (136). Additionally, FIV interacts with dendritic cells during the early stages of infection, enabling these cells to transfer the virus to susceptible T cells, thereby initiating a significant burst of viral replication (137). In addition to leukocytes, Feline Immunodeficiency Virus (FIV) has been shown to infect various cells in the central nervous system, including astrocytes and microglial cells. Infection in astrocytes impairs their ability to scavenge extracellular glutamate, while in microglial cells, FIV infection occurs during the subclinical phase and facilitates viral dissemination within brain tissue (138, 139).

Furthermore, megakaryocytes, stromal fibroblasts, and mononuclear cells in the bone marrow have also been reported to be infected by FIV in cats, where these cells could act as targets and reservoirs of the infection (140, 141). Since the primary mode of FIV transmission is biting, the salivary gland of cats could act as a reservoir of FIV during the early stages of infection. A study has reported the infection of epithelial cells of the interlobular duct of the salivary gland in cats (142). Besides cellular reservoirs, various tissues can also act as a reservoir of FIV in cats. Lymph nodes, spleen, thymus, gastrointestinal tract, reproductive tract, liver, brain, and bone marrow can all harbor FIV during the late, asymptomatic phase of infection (17, 143–146).

## 9 Anti-FIV drugs

Cats infected with retroviruses require specialized care and management, which, when provided, can enable them to live healthy lives for many years. Most retrovirus-infected cats are effectively managed through symptomatic therapy, while antiviral chemotherapy is recommended only in exceptional cases of FIV infections due to the limited proven efficacy and potential toxicity of antiviral drugs (147). The antiviral drugs commonly used in cats have been authorized for use in humans and are specifically designed to treat the infection caused by the human immunodeficiency virus (HIV) (147). Combination antiretroviral therapy (cART) has been reported to alleviate FIV-associated oral disease by maintaining the integrity of the oral mucosal microbiota in FIV-infected cats (148). However, many of these antiviral drugs have shown ineffectiveness in treating FIV in cats or cause adverse side effects (149). FIV-infected cats treated with zidovudine, a nucleoside reverse transcriptase inhibitor, which is used for the treatment of HIV, have been reported to be resistant to the long-term antiretroviral therapy (150). Additionally, fozivudine tidoxil, which is a lipid-zidovudine conjugate, has been reported to decrease plasma and cell-associated viremia during the first 2 weeks of infection but was ineffective in protecting cats from FIV infection, as all cats were infected by 6 weeks (151). Didanosine, another medication used for the treatment of HIV, has been reported to have antiviral activity against FIV *in vitro* and in animal studies but caused toxic neuropathy in cats (147, 152).

Other than the HIV antiviral drugs, various other strategies have been explored for the treatment of FIV. Various peptides have shown the ability to inhibit the replication of FIV in different feline cell lines (Table 2) (153, 154). Peptides 5–7, spanning amino acids E225 to P264 in a conserved region of the surface protein of the Petaluma isolate of FIV, effectively inhibited FIV-induced syncytium formation and suppressed viral replication in a time-dependent manner (153). Peptide 59, a 20-mer synthetic peptide derived from the membrane-proximal ectodomain of the FIV transmembrane glycoprotein, demonstrated the ability to inhibit the growth of tissue culture-adapted FIV in feline fibroblastoid CrFK cells (154). Similarly, RNA interference technology (lentiviral vector expressing a short hairpin RNA targeting the gag gene of FIV) has also shown the ability to inhibit FIV replication in cell lines that were chronically infected with FIV (155). Seetaha et al. reported the potential of crude extracts of different medicinal mushrooms to inhibit FIV reverse transcriptase *in vitro*, where ethanol extract from dried fruiting bodies of *Inonotus obliquus* and hexane extract from dried mycelium of *I. obliquus* showed the strongest inhibition with IC50 values of 0.80 ± 0.16 μg/mL and 1.22 ± 0.20 μg/mL, respectively (156). Derivatives of different compounds, such as 8-Difluoromethoxy-4-Quinolone, 1,2,3-dithiazole, and T140 derivatives (Table 2) (157, 158).

**Table 2:** FIV inhibition activity of different compounds.

**Compounds**	**Effects**	**References**
peptide/EGPTLGNWAREIWATLFKKA, LGNWAREIWATL, and TRQCRRGRIWKRWNETITGP from FIV gp95 protein	Inhibited replication of FIV in feline lymphoid cells and FIV-induced p25 production and syncytium formation	(153, 241)
peptide/LQKWEDWVRWIGNIPQYLKG from the membrane proximal ectodomain of FIV transmembrane glycoprotein	Inhibited replication of FIV in feline lymphoid cells	(154)
peptide/LGEWYNQTKELQQKFYEIIMNIEQNNVQVKKGLQQ from the C-terminal HR2 domain of FIV gp40 protein	inhibited the FIV replication and cell membrane fusion mediated by FIV-infected cells	(242)
Peptide/WEDWVGWI derived from the proximal external region of the FIV gp36 protein	Inhibited fusion of FIV with the host cell membrane	(243)
RNA interference/short hairpin RNA targeting the FIV gag gene	inhibited FIV replication in chronically infected feline T-lymphoid cell lines	(155, 244)
Human interferon-α	Increased survival of FIV-infected cats and ameliorated disease condition of FIV-infected cats	(245)
Recombinant feline interferon-ω	Caused clinical improvement of FIV-infected cats, showing antiviral property against FIV in infected cats	(160–162)
1,2,3-dithiazole derivative/(Z)-N-(4-Chloro-5H-1,2,3-dithiazol-5-ylidene)-3-methyl-1H-pyrazol-5-amine	Non-toxic to feline kidney cells and showed an antiviral effect against FIV with an effective concentration (EC) of 0.083 μM in feline embryonic fibroblast cells	(246)
1,2,3-dithiazole derivative/4-Phenyl-5H-1,2,3-dithiazole-5-thione	Non-toxic to feline kidney cells and showed an antiviral effect against FIV with EC of 0.023 μM in feline embryonic fibroblast cells	(247)
Epidithiodiketopiperazines derivative/Ethyl 3-((±)-(1S,4S)-5-benzyl-3,6-dioxo-7-sulfido-7-thia-2,5-diazabicyclo [2.2.1]heptan-2-yl)propanoate	Non-toxic to feline kidney cells and showed an antiviral effect against FIV with an EC of 0.053 μM in feline embryonic fibroblast cells	(248)
derivatives of T22, a peptide from horseshoe crab blood cells/CXC-Chemokine receptor 4 (CXCR-4) antagonists	Inhibited syncytium formation in cells expressing CXCR-4 in FIV-infected cells and the inhibited FIV replication in the feline lymphoid cell line	(249)
Bicyclam derivatives/plerixafor (CXCR4 antagonist)	Reduced viral load in naturally FIV-infected cats in comparison to placebo, but did not improve immunological and clinical variables associated with FIV infection	(163)
Reverse transcriptase inhibitors/didanosine, emtricitabine, and lamivudine	inhibited FIV replication in feline PBM cells at non-cytotoxic concentrations (10μM)	(250)
Pentathiepin derivative/6,7,8-trimethyl-7H-[1,2,3,4,5]pentathiepino[6,7-c]pyrrole	inhibited FIV replication at EC50 = 4 nM with low cytotoxicity to FIV kidney cells	(251)
Human immunodeficiency virus integrase inhibitors/L-870,810 (naphthyridine carboxamide)	inhibited FIV replication in the feline lymphoid cell line with EC= 2.4 nM and didn't show cytotoxicity up to 10 μM	(252)
Protease inhibitor/TL-3	Reduced viral load and eliminated FIV-induced alterations in the central nervous system of FIV-infected cats	(253)
Analog of anti_HIV drug tenofovir/(R)-9-(2-phosphonylmethoxypropyl)-2,6-diaminopurine	Reduced FIV viral load in the plasma of FIV-infected cats, improved wellbeing, and quality of life of FIV-infected cats, as measured by Karnofsky score	(254)
Avemar (fermented wheat germ extract)	Inhibited viral replication in FIV-infected MBM lymphoid cells and Crandell Rees feline kidney cells	(255)

Other therapies, such as the treatment of FIV-infected cats with recombinant human interferon-α, have also been explored. The recombinant human interferon-α therapy did not affect the function of the kidney or liver and ameliorated the clinical signs of FIV infection in cats naturally infected with FIV during the course of the treatment (159). Although the cats remained clinically healthy once the therapy was stopped, signs such as cytopenia and a reduction in CD4+/CD8+ worsened after the therapy discontinuation (159). Recombinant feline interferon omega was the first interferon approved for veterinary use in cats (160). Studies have demonstrated its potential to enhance innate immunity, leading to a reduction in clinical symptoms and co-infections in cats naturally infected with FIV (161, 162). While immunomodulators such as recombinant interferons (e.g., recombinant human IFN-α, recombinant feline IFN-ω) can alleviate clinical signs in FIV-infected cats (36, 37), their long-term use warrants careful monitoring to avoid potential immune dysregulation. The use of interferon can increase the risk of neutropenia, decreased blood counts, kidney dysfunction, infections, and concomitant diseases (36, 38, 39). To balance symptomatic relief with safety, a comprehensive monitoring protocol is essential, including periodic measurements of complete blood counts, thrombocytopenia, leukopenia, creatinine, creatine kinase, and liver function (39). In addition, regular clinical evaluations should assess the emergence of opportunistic infections or organ dysfunction. Employing such multi-parametric surveillance in long-term studies will help ensure that treated cats benefit from improved clinical management without unintended immune side effects.

Although some drugs reduce viral load in naturally FIV-infected cats, other drugs have been unsuccessful in providing protection to cats from acute FIV infection and in ameliorating the clinical and immunological signs of FIV infection in these cats (151, 163). Similarly, treatment with bee venom melittin has improved the general health status of FIV-infected cats but did not influence the immunological parameters such as the CD4/CD8 ratio and lymphocyte subpopulations (164).

Given the limited efficacy and tolerability of HIV-derived drugs in cats, future antiviral discovery for FIV should follow a feline-specific prioritization framework. The most promising candidates will combine a strong safety profile in cats with a high genetic barrier to resistance and broad activity across diverse FIV clades. Agents should demonstrate activity in primary feline lymphocytes and macrophages and show predictable pharmacokinetics suitable for long-term use. Equally important are resistance surveillance, cost-effective formulation for veterinary practice, and translational value from cross-lentivirus research, while ensuring host compatibility in cats.

## 10 FIV vaccines

According to the Vaccination Guidelines Group (VGG) of the World Small Animal Veterinary Association (WSAVA), the FIV vaccine is considered one of the non-core vaccines for pet cats (165). Significant efforts have been made to create a preventive vaccine for FIV, resulting in Fel-O-Vax FIV. This vaccine contains two strains of FIV—FIV Petaluma (subtype A) and FIV Shizuoka (subtype D)—and was approved for use in cats over 8 weeks old in Australia, Canada, and the USA (166, 167). A case-control field study showed that Fel-O-Vax, the only FDA-approved vaccine against FIV, has only a 56% protective rate among client-owned cats in Australia, raising doubt about the efficacy of the vaccine under field conditions (168). Similarly, another study has reported that the cats vaccinated with Fel-O-Vax did not generate broadly neutralizing antibodies, suggesting that Fel-O-Vax protection may not be effective against certain highly infectious recombinant strains of FIV that are currently circulating in Australia (169). Due to this, the Fel-O-Vax vaccine was removed from the market in the USA and Canada in 2017, although it remains available in Japan, Australia, and New Zealand. According to the WSAVA, it has never been licensed in Europe (165).

Researchers worldwide have been actively engaged in the development of vaccines against FIV (Table 3). However, FIV vaccines developed to date have shown limited effectiveness (168, 170, 171).

**Table 3:** FIV vaccines and their status.

**Vaccine**	**Effects**	**Phase of vaccine development**	**References**
Fel-O-Vax vaccine	No significant prevalence difference (vaccinated vs. unvaccinated) in cats.	Late-phase development	(168)
	The protective rate of the vaccine in client-owned cats (Australia) was 56%.		
Fel-O-Vax vaccine	Two vaccination regimes (primary vaccination: 3 doses, 2–4 weeks apart; annual vaccination: 1 dose/12 months) generated a significant antibody response against FIV gp24 and gp40	Late-phase development	(256)
	Anti-p24 and anti-gp40 antibodies are variably detectable 12 months post-vaccination		
	Stronger antibody response in the primary group vs. the annual group		
Fel-O-Vax vaccine	No protective effect of vaccination on FIV infection among vaccinated and unvaccinated domestic cats with outdoor access	Late-phase development	(257)
Fel-O-Vax vaccine	10/14 vaccinated cats were fully protected for 48 weeks against FIV subtype B strain infection, but 5/5 controls were persistently infected with FIV	Late-phase development	(258)
Fixed-cell virus vaccine (FIV-M2 strain fixed with paraformaldehyde)	None (*n* = 12) of the immunized cats had evidence of FIV infection	Late-phase development	(184)
	5 of 14 control cats were infected.		
	The vaccine was safe and immunogenic and did not transmit infection		
Recombinant canarypoxvirus-based FIV vaccine in combination with an inactivated FIV-infected cell vaccine	Induced FIV-specific CTL and humoral responses	Early-phase development	(259)
	All vaccinated cats were protected from homologous FIV challenge		
	Partial to full protection in vaccinated cats against a heterologous FIV infection given 8 months after the initial challenge		
Recombinant viral vector modified vaccinia virus Ankara (MVA) expressing the V1–V3 variable regions of the FIV-B envelope protein	Stimulation of cellular and humoral immune responses through interferon-gamma and antibody production	Discovery/feasibility phase	(177)
Autologous monocyte-derived dendritic cells loaded with an aldithriol-2-inactivated FIV isolate	Immunization-induced PBMC proliferation and antibody response to FIV	Early-phase development	(180)
	Infection rates post-FIV challenge in vaccinated cats were similar to those of control cats		
LMgag/pND14-Lc-env recombinant DNA vaccine (recombinant *Listeria monocytogenes*) expressing FIV Gag protein and delivering an FIV Env	The provirus was undetectable in all the analyzed tissues after 1 year of vaginal FIV challenge	Early-phase development	(260)
	High vaginal FIV-IgA in three vaccinated cats pre-challenge and in all five cats 1-year post-challenge		
FIV vaccine consisting of 500 μg inactivated whole virus (FIV_Pet_ plus FIV_Shi_) at a 50/50 ratio of each strain, supplemented with cytokine	Provided 40–100% protection rates against different FIV subtypes in cats	Early-phase development	(190)
Multi-antigenic peptide vaccine consisting of evolutionarily conserved epitopes between FIV and human immunodeficiency virus-1	Induced FIV-specific T-cell immunogenicity in vaccinated cats	Early-phase development	(183)
	15/19 (78.9%) vaccinated cats were protected against the FIV challenge		
Proviral DNA vaccine consisting of FIV provirus with a vif gene deletion coexpressing feline interferon-γ	Higher frequency of FIV-specific T-cell proliferation in immunized cats	Early-phase development	(181)
	Absence of antiviral antibodies after vaccination		
*vif*-deleted FIV provirus DNA in combination with feline tumor necrosis factor-α and granulocyte macrophage-colony stimulating factor expression plasmids	Increased T cell response specific to FIV compared to other vaccination regimes	Early-phase development	(261)
	Did not suppress viral loads post-challenge with the FIV-PPR isolate		

Classification of the developmental phase of the FIV vaccines is based on Francis (262).

Inactivated or attenuated FIV preparations, as well as FIV proteins administered with or without adjuvants, have demonstrated only limited efficacy in protecting against FIV infection in cats (171–174). Vector-based approaches have also been largely unsuccessful: Semliki Forest virus and modified vaccinia virus Ankara (MVA) vectors expressing FIV Rev and OrfA did not elicit sufficient cell-mediated and humoral immunity and were unsuccessful in protecting cats against FIV (175). Moreover, one study reported increased viremia in cats vaccinated with recombinant vaccinia virus expressing the FIV envelope protein incorporated into an immune-stimulating complex (176). Recently, Andrade et al. developed an MVA-based vaccine expressing the variable V1–V3 region of the FIV-B envelope protein, which successfully induced both cellular and humoral immune responses in mice. However, the efficacy of this vaccine candidate has not yet been evaluated in cats (177).

A vaccine based on purified feline immunodeficiency virus (FIV), incorporated into immune-stimulating complexes (ISCOMs), and recombinant FIV p24 ISCOMs successfully elicited antibodies against the core protein p24. However, it failed to produce virus-neutralizing antibodies and did not protect cats from FIV infection when challenged intraperitoneally with 20 infectious units of FIV. Notably, vaccinated cats became viremic earlier than their unvaccinated counterparts (171). Moreover, the ISCOM adjuvanted vaccine candidate containing FIV OrfA and Rev proteins also showed immunogenicity but could not protect cats from the FIV challenge (178). Similarly, other vaccine candidates have also shown ineffectiveness in providing protection against FIV (173, 174).

The attenuated FIV strain lacking ORF-A was tested as a vaccine candidate in nine cats challenged with wild-type FIV (179). Of the nine cats, only three were free from the challenge virus, and in the remaining vaccinated cats, CD4 lymphocyte counts and viral loads were either unaffected or changed only slightly and transiently (179). Similarly, dendritic cells loaded with inactivated FIV generated FIV-specific antibodies, but the frequency at which the challenge virus infected the vaccinated cats was similar to that in the control animals (180). Additionally, another vaccine candidate, an attenuated FIV in which the vif gene has been deleted and co-expressed interferon-γ, was also unable to provide protection to immunized cats from challenge with a biological FIV isolate (181).

Another vaccine candidate consisting of FIV CD134 or surface glycoprotein alone or in complex generated neutralizing antibodies against CD134 and surface glycoprotein, but it could not provide protection to vaccinated cats from FIV challenge (174). A DNA vaccine consisting of replication-defective FIV due to deletion of FIV integrase (ΔIN) or reverse transcriptase (ΔRT) genes provided protection to only 5/18 (ΔIN) and 2/12 (ΔRT) vaccinates challenged with a low virulence strain of FIV, i.e., FIV-Petaluma. However, rechallenging the cats protected from the FIV-Petaluma strain with the relatively more virulent FIV Glasgow-8 strain did not provide sterilizing immunity against the more virulent FIV strain (182). The vaccine candidates studied so far have not shown the desired effectiveness; hence, new avenues should be continuously explored for the development of novel vaccine candidates against FIV. One approach could be to develop multi-antigenic peptides consisting of immunogenic T cell and B cell epitopes as vaccine candidates. Sahay et al. designed a multi-epitope vaccine containing conserved T-cell epitopes from reverse transcriptase and p24 FIV proteins, which protected 78.9% of vaccinated cats from FIV challenge (183).

## 11 Adjuvants used in FIV vaccines

Several experimental FIV vaccine candidates have been developed and tested in cats, some of which have included adjuvants to improve their effectiveness. In one study, dendritic cells were used as live adjuvants to improve the immune response of a whole-inactivated FIV vaccine (180). Animals that received the vaccine showed obvious signs of increased peripheral blood mononuclear cell proliferation and antibody titers specific to FIV following immunization. Nevertheless, the challenge of vaccinated cats with the homologous virus was unsuccessful in providing protective immunity against the infection and further reduced CD4+T lymphocyte numbers in the vaccinated cats post-challenge (180). Another study has used alum, an adjuvant that has been allowed for use in cats, but the formulated vaccine did not protect cats from FIV infection (174). Similarly, recombinant protein vaccines with ISCOM adjuvant were also unsuccessful in protecting cats from the FIV challenge (178). The use of incomplete Freund's adjuvant in a fixed-cell FIV vaccine and adenyl-muramyl dipeptide adjuvant in a fixed-cell dual-subtype FIV vaccine has also been reported in FIV vaccine candidates (184, 185). In some vaccine candidates against FIV, adjuvants have not been used (175, 177, 186, 187). One experimental FIV vaccine, which is approved for use in cats, known as the Fel-O-Vax FIV vaccine, contains an oil emulsion adjuvant called Fort Dodge-1 adjuvant (188–191). A multi-antigenic peptide vaccine candidate against FIV has also been used with Fort Dodge-1 adjuvant (183).

Different DNA vaccines against FIV have used molecular adjuvants, including IL-18 DNA, IL-12 plus IL-18 DNA, and IFN-γ (181, 182, 188, 192). However, these vaccines were also not very effective in providing protection from the FIV challenge in cats (188). The inclusion of IFN-γ DNA adjuvant did not enhance the efficacy of immunization with a DNA vaccine (FIV-pPPΔvif DNA) (181). In another study, DNA vaccination with an IFN-γ DNA adjuvant did not protect cats from post-challenge FIV infection (193). Cats immunized with a DNA vaccine (FIV_GL8_ΔRT), along with IL-12 plus IL-18 DNA or IL-12 alone, did not produce antiviral antibodies and showed a reduction in virus-specific CTL activity. However, immunization with a viral DNA (FIV_GL8_ΔIN) with an IL-18 DNA adjuvant induced virus-specific CTL activity. Nevertheless, both DNA vaccines, FIV_GL8_ΔRT and FIV_GL8_ΔIN, with cytokine DNA adjuvants, were not able to provide sterilizing immunity against the virulent FIV strain challenge post-vaccination in cats (182). Vaccination with an FIV DNA vaccine containing FIV gp140 DNA along with feline IL-12 DNA showed that the addition of IL-12 DNA significantly enhanced the response against FIV, and this vaccine protected three out of four cats from challenge infection (192). Similarly, cats immunized with an FIV gp140 DNA vaccine along with feline IL-16 or feline cytosine phosphoguanosine (CpG) had less proviral DNA in PBMCs and became less viremic after FIV challenge infection compared to cats vaccinated only with FIV gp140 DNA, suggesting the potential of IL-16 and CpG as possible adjuvants in FIV vaccines (194).

Overall, there is only one licensed FIV vaccine available that uses an adjuvant. The development and testing of experimental FIV vaccines with adjuvants suggest that adjuvants may be an important component in the development of an effective FIV vaccine. Further research is needed to determine the most effective adjuvant strategies for FIV vaccines. In future adjuvant system families, such as AS01, AS03, AS04, MF59, and CpG, which are used in other licensed or nearly licensed virus vaccine candidates, can be tested as adjuvants for FIV vaccines (195, 196). Furthermore, the adjuvant potential of a new adjuvant called Matrix-M, containing fraction-A and fraction-C of *Quillaja saponaria* Molina extract and used in a COVID-19 vaccine authorized by the European Medicines Agency, can be explored for developing FIV vaccines (197). AS01 and AS04 contain MPL (3-deacylated monophosphoryl lipid), a toll-like receptor-4 (TLR-4) agonist.

Similarly, other adjuvants that can activate other TLR molecules, such as TLR-7 and TLR-8, should also be analyzed for FIV vaccines. In this regard, Alhydroxiquim-II, an adjuvant used in the COVID-19 vaccine COVAXIN, can be used.

## 12 Immunoinformatics on FIV research

Immunoinformatics has recently been employed in the development of viral agent vaccines, involving the application of computational methods and resources to study immune system functions. Immunoinformatics enables the precise storage and analysis of immune-related data, facilitating a deeper understanding of immune system mechanisms and functions, which in turn helps in the development of vaccines (198). Epitope-based vaccines assisted by computational tools, derived from viral immunodominant antigens, have been used to develop vaccine alternatives since their activation of helper CD4+ T cells, CD8+ cytotoxic T cells, and B cell activation through helper T cells, allowing them to differentiate into plasma cells that produce antibodies, which are essential for the complete clearance of viruses from the host (199). Although there is no significant research based on the immunoinformatics approach for FIV, different studies have employed this approach to advance new vaccines for veterinary viral agents, such as canine distemper virus (200), canine circovirus (201), canine parvovirus (202), and FIPV (203), among others. Moreover, immunoinformatics has been utilized in the context of HIV to explore T- and B-cell epitopes based on genomic information and antigenic proteins, such as gp120 (204–206). Thus, further studies must be conducted to develop a new generation vaccine based on computationally predicted multiple epitopes for FIV.

## 13 Future perspectives

High-throughput genomics, transcriptomics, and proteomics in cats could provide valuable insights into the signaling pathways and molecular mechanisms underlying FIV infection. These approaches may also reveal immune-modulatory pathways and novel biomarkers, thereby facilitating the development of targeted therapies and the discovery of new drug targets.

Recent advances in gene-editing technologies, particularly CRISPR/Cas systems, offer exciting opportunities for the control of FIV. CRISPR-Cas systems hold promise for reducing the proviral load of FIV by suppressing viral transcription and limiting the production of infectious virions and potentially achieving a functional cure (207). Such strategies have been investigated in HIV research with promising outcomes (208), and adapting similar approaches for FIV could open novel therapeutic avenues. Beyond proviral excision, CRISPR-based tools can also be used to edit host factors and co-receptors critical for viral entry to generate resistance in susceptible feline cells (209). In addition to therapeutic applications, gene-editing techniques hold potential for vaccine development. CRISPR/Cas can accelerate the design of attenuated or replication-deficient viral strains that serve as safe and immunogenic vaccine candidates. These strategies may overcome the limitations of conventional vaccine platforms, which have shown inconsistent efficacy in cats.

The development of antiviral drugs for FIV remains an underexplored area compared to HIV research, despite the structural and pathological similarities between the two viruses. Future efforts should prioritize drug discovery targeting conserved viral proteins, which are essential for FIV replication and represent viable therapeutic targets. Structure-based drug design, aided by advances in crystallography, molecular docking, and molecular dynamics simulations, can accelerate the identification of small-molecule/natural compound inhibitors with high specificity and low toxicity (210).

mRNA-based vaccine approaches also represent a promising avenue for FIV prevention. Unlike traditional inactivated or recombinant protein vaccines, mRNA vaccines can be rapidly designed to encode multiple conserved FIV antigens and delivered using lipid nanoparticles, eliciting both strong humoral and cellular immune responses. The success of mRNA platforms against emerging human viral pathogens, such as SARS-CoV-2 (211, 212), demonstrates their flexibility and scalability; similar strategies could be adapted for veterinary use. For FIV, mRNA vaccines encoding Env and p24 epitopes, combined with potent adjuvant systems, may overcome limitations of past vaccines by inducing broader, durable, and cross-clade immunity in cats. Additionally, viral vector–mediated mRNA vaccines can be optimized to enhance immunity in cats against FIV. Future research should focus on selecting optimal viral vectors (e.g., adenoviral, modified vaccinia Ankara, or lentiviral platforms) (213), assessing safety and long-term immunogenicity in cats, and evaluating efficacy against diverse FIV subtypes in both experimental and natural challenge models. If successful, viral vector–delivered mRNA vaccines may represent a new generation of FIV vaccines with the potential to overcome the limitations of earlier approaches.

Multi-epitope vaccines represent a promising strategy for overcoming the limitations of conventional FIV vaccines, which often provide incomplete or strain-specific protection. By combining conserved B-cell and T-cell epitopes from multiple viral proteins, such as Env, Gag, and p24, these vaccines can elicit broader and more durable immune responses across diverse FIV subtypes. Advances in bioinformatics and immunoinformatics now make it possible to predict and design epitope combinations with high immunogenic potential while minimizing off-target effects. Incorporating these epitopes into delivery systems, such as nanoparticles, viral vectors, or DNA/mRNA platforms, may further enhance immunogenicity and the longevity of protection. In addition, multi-epitope vaccines offer the flexibility to target both humoral and cellular arms of the immune system. By incorporating epitopes from multiple viral proteins, such vaccines can elicit broad and robust immune responses while addressing major challenges in FIV vaccine design, including antigenic shifts, antigenic drifts, and genetic variability among viral strains (214). This strategy enhances the likelihood of cross-protection against diverse FIV subtypes and reduces the risk of immune escape, which has historically hindered the success of conventional vaccines. Future work should focus on the experimental validation of *in silico*–designed epitope constructs, optimization of adjuvant formulations, and evaluation of cross-protection against circulating FIV strains in natural populations. If successful, multi-epitope vaccines may provide a next-generation solution with greater global applicability and efficacy in FIV prevention.

Additionally, comprehensive molecular epidemiology studies are required to characterize regional FIV variants, evaluate the cross-protection of vaccines, and assess the role of viral recombination in vaccine escape. Such studies will ensure that future vaccines and therapeutics are effective across a broad range of viral subtypes. A critical future goal in FIV vaccine research is the development of formulations capable of conferring protection against the predominant circulating subtypes, as well as the circulating recombinant forms (190). Given the high genetic diversity and recombination potential of FIV, next-generation vaccine strategies must focus on inducing broad-spectrum and durable immunity that remains effective across both subtype-specific and recombinant viral strains. To overcome this, future vaccine strategies should prioritize the inclusion of conserved epitopes from across circulating subtypes, identified through immunoinformatics and comparative genomics, to maximize cross-protection. In addition, multivalent formulations may help broaden immune responses against diverse strains. Multivalent vaccines offer an advantage over monovalent approaches by inducing polyclonal antibody responses against multiple FIV variants in a single formulation, thereby providing broader protection against recombinant and emerging subtypes (215).

Much of the current understanding of FIV comes from experimental infections in controlled laboratory settings. While these studies have provided valuable insights into viral pathogenesis and immune dysregulation, they may not fully capture the variability observed in naturally infected cats. In real-world conditions, prevalence and disease progression are influenced by multiple factors, including co-infections, environmental stressors, nutritional status, and host genetic background. As a result, naturally infected cats often display a broader range of clinical outcomes, including milder or atypical manifestations compared to laboratory models. For example, some FIV-positive cats remain asymptomatic for life, whereas others develop severe opportunistic infections or neoplasia.

Additionally, evidence from naturally infected cats also underscores the importance of considering real-world conditions when interpreting laboratory data. For example, outdoor FIV-positive cats have been reported to show more pronounced hypergammaglobulinemia and elevated total protein levels compared to indoor cats, likely reflecting greater antigenic exposure and co-infections in outdoor environments (31). Such findings illustrate how environmental and lifestyle factors shape immune responses and clinical manifestations in naturally infected cats, in contrast to the more uniform outcomes observed in controlled laboratory infections. Moreover, it remains difficult to directly compare treatment outcomes between experimentally infected cats maintained under laboratory conditions and pet cats naturally infected with diverse FIV field strains. This discrepancy underscores the importance of real-world evidence. While laboratory models provide controlled insight into mechanisms and drug activity, only studies in naturally infected cats can capture the influence of co-infections, environmental stressors, and viral diversity. Therefore, future progress will depend on well-designed, double-blinded, placebo-controlled clinical trials in naturally FIV-infected cats to rigorously determine the efficacy, safety, and long-term tolerability of novel antiviral compounds (32). These complexities underscore the need to interpret experimental data with caution and highlight the importance of integrating findings from naturally infected populations to obtain a more accurate picture of FIV epidemiology and clinical impact. Future research should therefore prioritize longitudinal studies in naturally infected cats, which will be critical for validating laboratory findings, refining vaccine efficacy, and guiding therapeutic interventions under real-world conditions.

Future research should also aim to overcome the geographical bias evident in current FIV studies, which are predominantly concentrated in developed countries such as Australia, the USA, and Japan. Expanding epidemiological surveys and vaccine evaluations in Africa, Southeast Asia, and Eastern Europe will be essential to generate more representative data on prevalence and vaccine efficacy. Addressing this gap will require investment in diagnostic infrastructure, capacity-building initiatives, and targeted funding to support local research. International collaborations and global data-sharing platforms can further strengthen surveillance and ensure that findings are globally applicable, ultimately improving strategies for FIV prevention and control.

## 14 Conclusion

FIV is prevalent among both stray and household domestic cats throughout the world. FIV infects a wide variety of cells and causes mild to severe clinical signs, whereas some cats may not show any signs at all. FIV infection has been associated with neoplastic diseases, neurological dysfunctions, and renal diseases. Environmental factors, host characteristics, and genetic variations may influence the clinical signs and pathogenesis of FIV. The variation in genomic sequences and the presence of different subtypes of FIV pose challenges in the development of an effective vaccine. Most vaccine candidates have shown poor efficacy, and although most challenge studies with a commercially available dual-subtype FIV vaccine have shown satisfactory efficacy against FIV infection in controlled settings, its effectiveness in real-world situations still needs to be established.

Similar to the development of FIV vaccine candidates, several factors have also hindered the development of antiviral drugs against this disease. FIV has shown resistance to some antiretroviral drugs used for the treatment of HIV, while other HIV antiviral drugs could be toxic and ineffective in cats. *In vitro* studies have shown the potential of derivatives of different compounds, peptides, and interferon as antivirals against FIV, but their efficacy has not been determined in FIV-infected cats in properly designed trials. Furthermore, it is uncertain if the findings of laboratory experiments on infected cats regarding FIV antiviral drugs and vaccines can be applied to pet cats infected with naturally occurring strains of the virus. Due to this uncertainty, it is crucial to conduct more carefully planned trials that are double-blind and placebo-controlled in the future. These trials should involve naturally infected cats with retroviruses, and different antiviral compounds should be studied to determine their effectiveness and any potential adverse effects. Moreover, in the future, the potential of multi-epitope vaccines in protecting cats from FIV infection can be explored. Furthermore, future research is required to identify the best adjuvants that can be used in FIV vaccines, including the immunoinformatic approach.

Additionally, bridging molecular insights with clinical management is essential for improving the care of FIV-positive cats. From a veterinary perspective, clinical decision-making should emphasize early detection, ongoing monitoring, and preventive management in multi-cat environments. Routine monitoring—including physical examinations, complete blood counts, serum biochemistry, and analysis of the CD4^+^/CD8^+^ T-cell ratio—can help track disease progression and guide timely interventions. Molecular markers such as proviral load and viral RNA quantification, although currently more common in research, may eventually serve as adjunct diagnostic tools in practice for risk stratification. In multi-cat households, segregating FIV-positive cats from aggressive or uninfected cats, neutering to reduce fighting behavior, and testing all cats before group housing remain best practices for minimizing transmission. By integrating molecular knowledge with structured monitoring and practical management guidelines, veterinarians can make informed decisions that balance long-term health outcomes with the quality of life in FIV-infected cats.
